# Post-traumatic stress disorder symptom severity, trauma load and sleeping difficulties in trauma-exposed, treatment-seeking adolescents

**DOI:** 10.4102/sajpsychiatry.v24i0.1300

**Published:** 2018-09-20

**Authors:** Carissa van Aarde, Jani Nöthling, Cherie Armour, Soraya Seedat

**Affiliations:** 1 Department of Psychiatry, Stellenbosch University, South Africa; 2 Faculty of Life and Health Sciences, Ulster University, United Kingdom

## Abstract

**Introduction:**

Sleep disturbances are associated with various anxiety- and trauma-related disorders, and specifically with post-traumatic stress disorder (PTSD). Two of the core symptoms of PTSD are recurrent distressing dreams about the traumatic event and difficulty in falling or staying asleep (insomnia). Sleep is essential for functioning, with poor sleep compromising cognitive, emotional and behavioural regulations. Sleep is also of particular importance for brain development and information processing in children and adolescents. The aim of this study was to determine if PTSD symptom severity and trauma load is associated with nightmares and insomnia in a sample of trauma-exposed, treatment-seeking adolescents.

**Methods:**

A total of 358 South African adolescents, between 12 and 18 years of age, exposed to at least one DSM-5 qualifying traumatic event, were assessed for PTSD-related sleep difficulties using the Kiddie Schedule for Affective Disorders and Schizophrenia (KSADS). Childhood exposure to 10 common trauma types was also recorded using the KSADS trauma checklist. PTSD symptom severity was measured using the child PTSD checklist (CPC).

**Results:**

Adolescents reporting current trauma-related nightmares (*F*[268.2] = 0.68, *t* = -8.16, *p* = 0.000) and current insomnia (*F*[265.2] = 0.16, *t* = -9.03, *p* = 0.000) had significantly higher PTSD symptom severity scores compared to those without current nightmares and insomnia. Adolescents with current trauma-related nightmares (*F*[355.2] = 0.15, *t* = -3.30, *p* = 0.001) and insomnia (*F*[353.2] = 0.15, *t* = -2.51, *p* = 0.013) were also exposed to a significantly greater number of different trauma types compared to those without current nightmares and insomnia.

**Conclusion:**

Assessing and treating sleep difficulties related to PTSD in trauma-exposed adolescents, in an effort to reduce the developmental impact of trauma on the brain and general functioning, should not be overlooked. Longitudinal studies may contribute to a better understanding of the long-term effects of trauma-related insomnia and nightmares on mental and physical health outcomes.

